# Case Report: A Frameshift Mutation in MSH2 Exon 2 in a Kidney Recipient With Muir–Torre Syndrome

**DOI:** 10.3389/fonc.2021.681780

**Published:** 2021-06-24

**Authors:** Yifei Feng, Jianqing Feng, Jianrong Bao

**Affiliations:** ^1^ Department of Dermatology, The First Affiliated Hospital, Nanjing Medical University, Nanjing, China; ^2^ Department of Dermatology, Taicang Hospital of Traditional Chinese Medicine, Suzhou, China; ^3^ Department of Pathology, Taicang Hospital of Traditional Chinese Medicine, Suzhou, China

**Keywords:** Muir–Torre syndrome, sebaceous carcinoma, kidney transplantation, mismatch repair gene, next-generation sequencing, sigmoid adenocarcinoma

## Abstract

Muir–Torre syndrome (MTS), a rare subtype of Lynch syndrome, is mostly autosomal dominant, which is caused by germline mutations in DNA mismatch repair (MMR) genes, the resulting microsatellite instability (MSI) of which increases the risk of developing sebaceous and other visceral tumors. Several reports have showed an association between immunosuppressive agents and the progression of latent MTS. In this report, we described a 41-year-old man with a history of kidney transplantation, having a rapid growth of the nodule on the anterior chest under immunosuppressive therapy, which was histologically proved to be sebaceous carcinoma. Systemic evaluation for visceral malignancies revealed sigmoid adenocarcinoma. These findings were consistent with the clinical diagnosis of MTS. Histological findings showed an absence of MMR proteins, including MSH2 and MSH6 both in the sebaceous carcinoma and sigmoid adenocarcinoma on immunohistochemical (IHC) analysis. A frame-shift mutation of c.229_230delAG (p. Ser77fs) in the MSH2 exon 2 in the lesion was detected by next-generation sequencing (NGS) analysis. This case report not only reveals a new site of MSH2 mutation in this family of East Asian descent but also highlights the importance of adequate diagnosis for Muir–Torre syndrome, as well as further prevention of the development of latent visceral tumors in kidney transplant recipients.

## Introduction

Muir–Torre syndrome (MTS) is a rare subtype of Lynch syndrome caused by autosomal dominant mutations in DNA mismatch repair genes. Clinical diagnoses of MTS are made by the synchronous or metachronous occurrence of at least one sebaceous skin tumor and at least one visceral tumor (1). The most common tumors in MTS patients are sebaceous adenomas, which are often used as a skin marker to diagnose MTS; in contrast, sebaceous carcinomas are less common. Several lines of evidence suggest that immunosuppressive agents accelerate the progression of latent MTS; however, further research is required to better understand the complex linkages between them.

Herein, we describe a kidney transplant recipient with MTS at a new mutation site. Under immunosuppressive therapy, the patient acquired rapid growth of a dark-red nodule on the anterior chest, which was proven to be sebaceous carcinoma. A whole-body examination revealed that the patient also had sigmoid adenocarcinoma. Immunohistochemical (IHC) analysis showed diminished MMR protein expression, including MSH2 and MSH6 in both the sebaceous carcinoma and the sigmoid adenocarcinoma. Furthermore, we identified a novel frame-shift mutation of c.229_230delAG (p. Ser77fs) in the *MSH2* exon 2 in this family of East Asian descent. These findings assisted our understanding of the role of systemic evaluation for visceral tumors in MTS patients, especially those with a history of transplantation. They further implied a possible role of immunosuppressive therapy in MTS progression.

## Case Description

**Figure 1:**
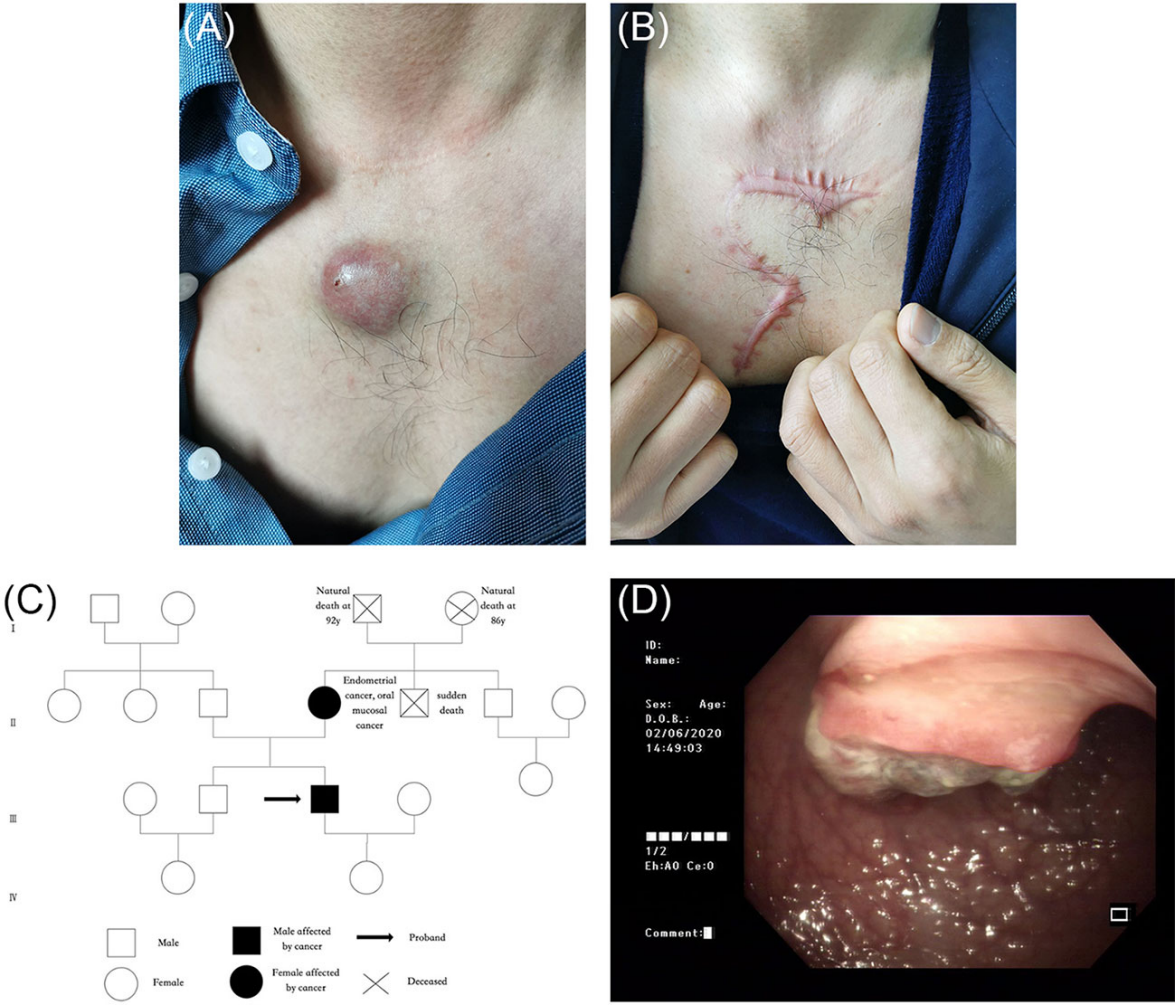
**(A)** A dark-red hard nodule of 3 cm * 3 cm * 2.5 cm in size was found on the anterior chest, with an opening at the edge. **(B)** Postoperative scar. **(C)** The pedigree chart of the index patient (black arrow) and his family. The patient’s mother had endometrial and oral mucosal cancer. **(D)** Colonoscopy showed irregular neoplasms protruding into the intestinal lumen 28 cm away from the anus, with the size of 3 cm * 3 cm whose irregular protrusions in the central part were brittle, hard texture, contact bleeding and covered with soiled moss.

**Figure 2:**
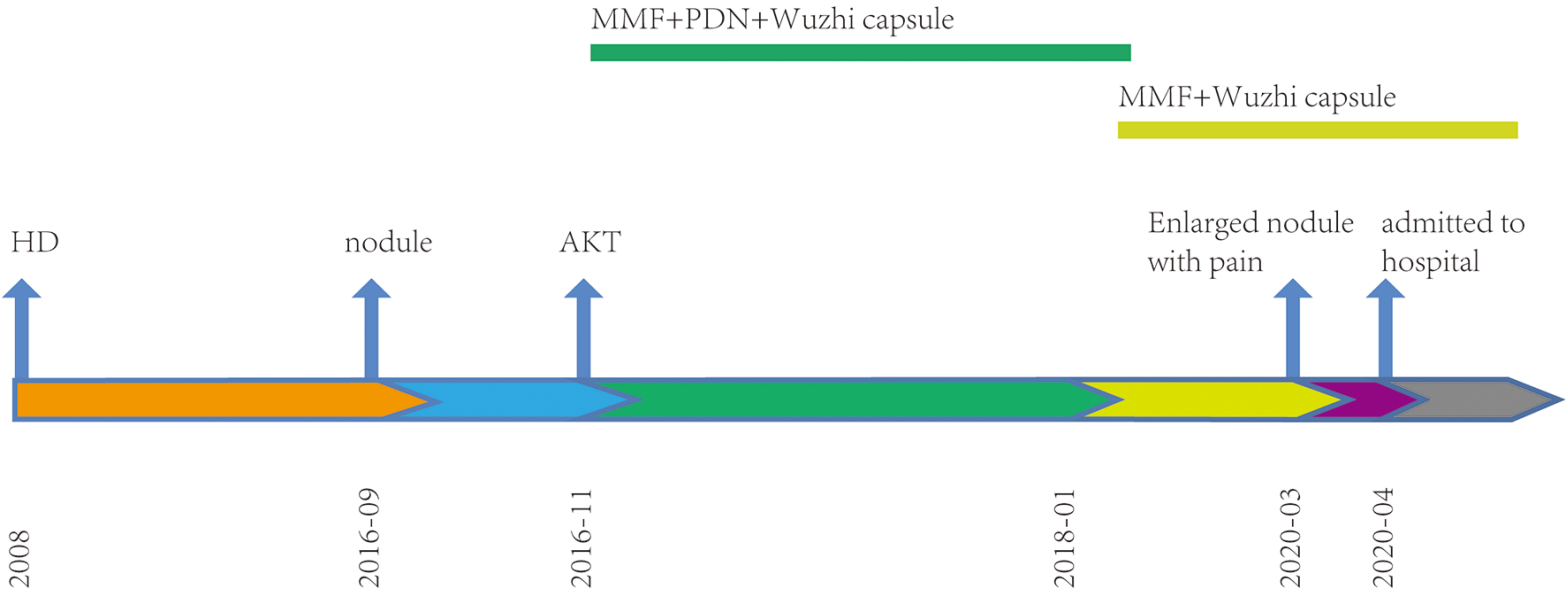
Timeline. Treatment procedures since renal therapy. HD, hemodialysis; AKT, allograft kidney transplantation; MMF, mycophenolate mofetil; PDN, prednisone.

In 2020, a 41-year-old man was admitted to the dermatology clinic at the Taicang Hospital of Traditional Chinese Medicine for a dark-red nodule on the anterior chest that had been present for 4 years. Over the past month, the major axis had rapidly increased from 1 to 3 cm, causing pain. Physical examination revealed a hard, dark-red nodule of 3 × 3 × 2.5 cm on the anterior chest that had an opening along an edge (**Figure 1A**). No swelling of superficial lymph nodes was found. The patient had been undergoing hemodialysis triweekly for chronic renal failure since 2008 and received allogeneic kidney transplantation in November 2016. His allograft kidney was maintained by immunosuppressive therapy comprising tacrolimus, mycophenolate mofetil, prednisone, and Wuzhi capsule, which was used to reduce serum glutamic-pyruvic transaminase (**Figure 2**). The family history of the patient included his mother, who had endometrial and oral mucosal cancer (**Figure 1C**).

**Figure 3:**
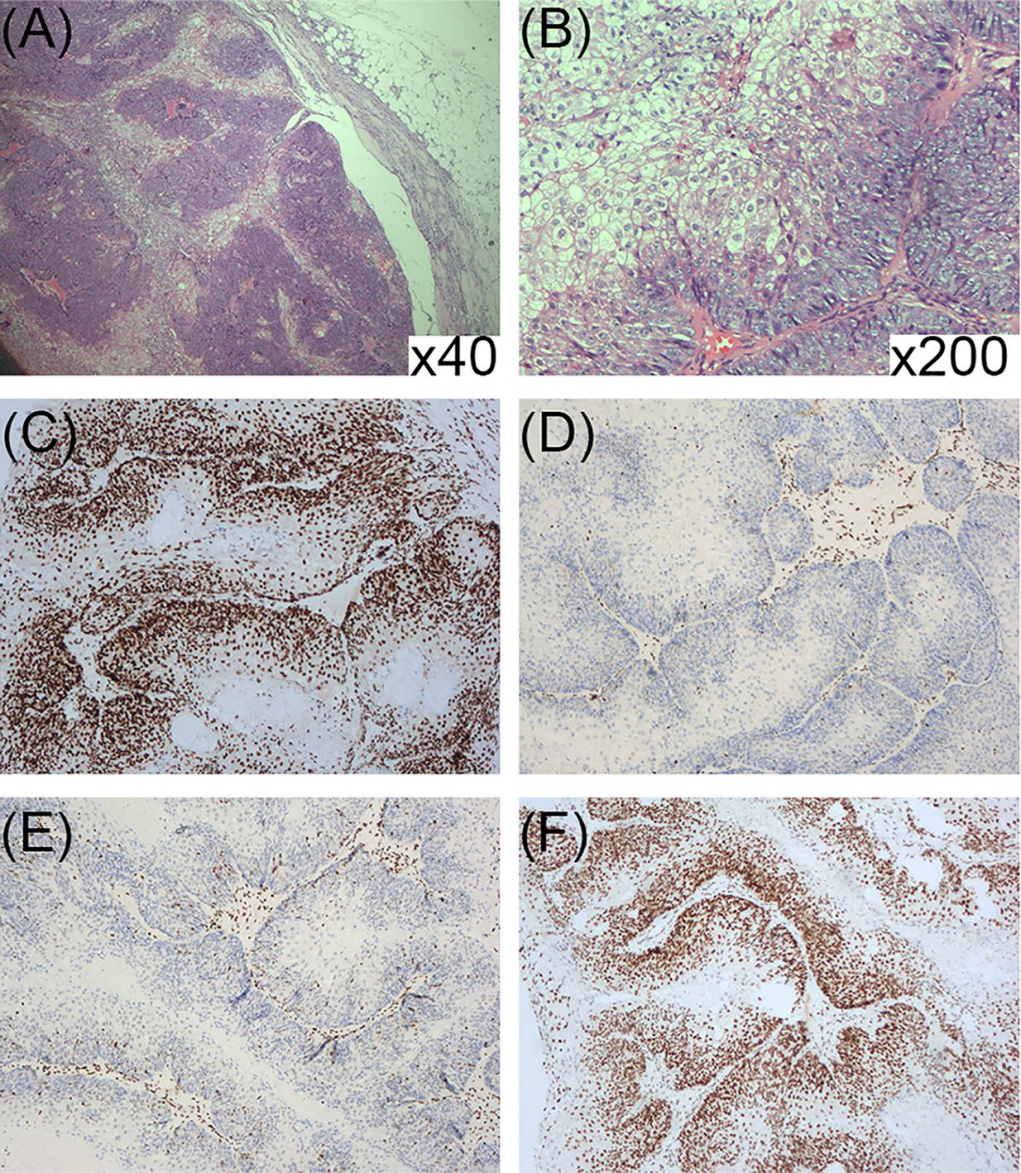
**(A, B)** Pathological presentation of sebaceous carcinoma on the chest: **(A)** Tumor cells formed nests or lobules of different sizes, with clear boundary and capsule (HE ×40). **(B)** The tumor was composed of basal-like cells and vacuolated sebaceous cells, some of which had large, hyperchromatic nuclei (HE ×200). **(C–F)** Immunohistochemical staining for DNA mismatch repair proteins (MMR proteins MLH1, MSH2, MSH6, and PMS2) of the sebaceous carcinoma; **(C)** Normal nuclear expression of MLH1 protein. **(D)** Lack of nuclear expression of MSH2 protein. **(E)** Lack of nuclear expression of MSH6 protein. **(F)** Normal nuclear expression of PMS2 protein.

Surgical resection of the tumor was performed for diagnosis and treatment (**Figure 1B**). Histopathology showed that tumor cells had formed nests or lobules of different sizes, with capsular and clear boundaries, presenting an infiltrating growth form that was characterized by the gradual differentiation from basal-like cells in the periphery to vacuolated sebaceous cells in the center. Tumor cells with large, hyperchromatic nuclei and increased mitotic figures formed large cell clusters, in which squamous epithelial differentiation and keratinization were seen. Small nests or single cancer cells had invaded the stroma, which was accompanied by an inflammatory response and granuloma (**Figures 3A, B** **)**. IHC findings were ADRP (+), AR (+), Ber-EP4 (−), CEA (−), CK5/6 (+), CK7 (−), EMA (+), Ki-67 (40–50%+), and P53 (few+). These findings were consistent with low-grade sebaceous carcinoma. IHC also showed complete loss of MSH2 and MSH6 expression but normal MLH1 and PMS2 expression (**Figures 3C–F**). Then, next-generation sequencing (NGS) was performed to test the four MMR genes in the peripheral blood, and a class 5 pathogenic frame-shift mutation of c.229_230delAG, p. Ser77fs in *MSH2* exon 2 was detected, with an allele frequency of 46.78%.

**Figure 4:**
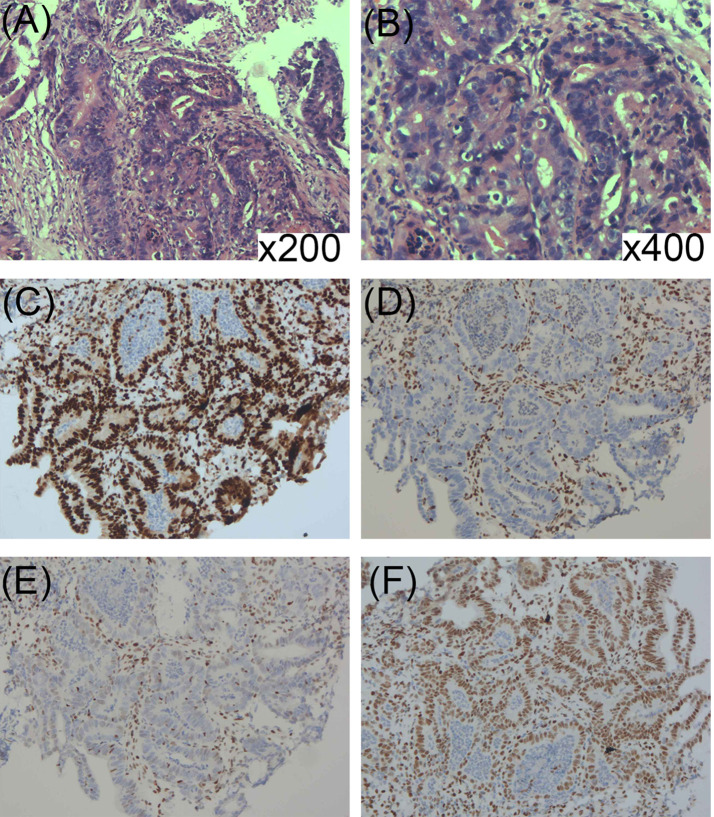
**(A, B)** Histopathology showed that tumor cells were arranged in columnar and glandular tubular shape, with obvious cellular atypia as well as interstitial and muscularis mucosae infiltrate (HE ×200; ×400). **(C–F)** Immunohistochemical staining for DNA mismatch repair-related proteins (MMR proteins MLH1, MSH2, MSH6, and PMS2) of the sigmoid adenocarcinoma: **(C)** Normal nuclear expression of MLH1 protein. **(D)** Lack of nuclear expression of MSH2 protein. **(E)** Lack of nuclear expression of MSH6 protein. **(F)** Normal nuclear expression of PMS2 protein.

Further screening for visceral tumors was conducted. Colonoscopy showed irregular neoplasms protruding into the intestinal lumen 28 cm away from the anus. The neoplasms had a size of 3 × 3 cm and irregular protrusions in the central region were brittle, hard, exhibited contact bleeding, and were covered with soiled moss (**Figure 1D**). An open radical sigmoidectomy was then performed. Histopathology showed that tumor cells were arranged in a columnar and glandular tubular shape, with obvious cellular atypia, as well as interstitial and muscularis mucosae infiltrate (**Figures 4A, B**). IHC findings were CAM5.2 (+), D2-40 (−), CD34 (−), Topo II (60%), P53 (60%), and Ki-67 (80%), suggesting a diagnosis of sigmoid adenocarcinoma. Furthermore, IHC also showed complete loss of MSH2 and MSH6 expression but normal MLH1 and PMS2 expression (**Figures 4C–F**). Together, these findings supported the diagnosis of MTS. The patient and his first-degree relatives underwent follow-up tumor monitoring. A few months later, his mother developed moderately differentiated squamous cell carcinoma from the mucosa of the left cheek, whose NGS results showed a frame-shift mutation of c.229_230delAG, p. S77Cfs*4 in *MSH2*, with a variant allele frequency of 42.01%.

## Patient Perspective

After recalling as much as possible, I found that before kidney transplantation, I already had a nodule growing on my chest. I didn’t care much at first, but recently it grew rapidly with pain, so I came to see a doctor. After a biopsy, the doctor made me do gene sequencing and visceral tumor screening with great foresight and finally made the diagnosis. In this process, the doctor introduced the manifestations and risks of Muir–Torre syndrome to me and my family in detail, and now I knew how to follow up on the occurrence of visceral tumors and deal with them in time.

## Discussion

MTS is a mostly autosomal dominant, rare subtype of Lynch syndrome characterized by an association with sebaceous skin tumors and visceral tumors. MTS is caused by hereditary mutations in DNA MMR genes including *MLH1*, *PMS2*, *MSH2*, and *MSH6*, deficiencies of which result in microsatellite instability (MSI). Nevertheless, approximately 33% of MTS patients do not exhibit MSI, and they comprise a subtype of MTS called MTS II (35%), which is mostly autosomal recessive (2).

Mutations in *MSH2* are the most common cause of MTS, accounting for 90% of cases (3) and, to date, several *MSH2* mutation sites have been detected worldwide. This case is the second report of MTS in China, and in contrast to other reports in the literature, a novel frame-shift mutation of c.229_230delAG (p. Ser77fs) in exon 2 of *MSH2* (NM_000251.2) was detected in this family of East Asian descent.

The use of immunosuppressive agents after organ transplantation has been found to accelerate skin tumor development in both MTS patients (**Table 1**) and individuals without a cancer predisposition. For MTS patients, this is probably associated with MSI and loss of MSH2 expression. Calcineurin inhibitors (CNIs), including cyclosporine and tacrolimus, are proven to promote tumor invasion, progression, and metastasis by increasing the activity of transforming growth factor *β* (TGF-*β*) and interleukin (IL)-6, as well as by inhibiting the nucleotide excision repair (NER) pathway (9). Additionally, the cytotoxic effects of azathioprine have been postulated to be ineffective in MMR defective cells (10), and its use plays a role in selecting the mutator phenotype that predisposes patients to develop sebaceous neoplasms (11). In comparison, mTOR inhibitors, including sirolimus and everolimus, have been proven to decrease the incidence of cutaneous malignancies by inducing G1 arrest, inhibiting TGF-*β* (12), inducing VEGF-related antiangiogenic activity and decreasing endothelial cell responsiveness to stimuli through VEGF receptors (13). A previous report described that changing from tacrolimus to sirolimus significantly decreased the formation of cutaneous neoplasms in a kidney transplant recipient with unrecognized MTS (4).

**Table 1:** Reports of Muir–Torre syndrome after transplantation.

Authors	Gender/Age	History	Medication history	Family history	Clinical manifestation	Laboratory examination	Diagnosis	Treatment	Prognosis
Levi, Z. et al. (4)	M/55	Sigmoid colon cancer (resection), transitional cell carcinoma of the renal pelvis (right nephroureterectomy, followed by kidney transplantation)	Tacrolimus, mycophenolate mofetil, prednisone	A family history of unexplained chronic hematuria resulted in renal failure of mother and daughter; a sister suffered from malignant melanoma	Multiple verrucous lesions on the face and chest	Germ-line mutation analysis: hMSH-2 (R680X)	Sebaceous adenoma, sebaceous carcinoma, keratoacanthoma, basal cell carcinoma, intestinal adenomatous polyp (consistent with MTS diagnosis)	Change from tacrolimus to sirolimus	No new sebaceous adenoma
Landis, M.N. et al. (5)	M/52	Kidney transplantation for malignant hypertension, hepatitis C	Tacrolimus, mycophenolate mofetil, prednisone	The father, a brother, three paternal uncles, and a paternal cousin had colon cancer	Several skin-colored umbilical papules on face and scalp, one intestinal polyp	IHC: MSH-2(−); Germ-line mutation analysis: MSH2 (34_35insG)	Sebaceous gland neoplasms, sebaceous adenoma, sebaceous carcinoma, basal cell carcinoma	Mohs surgery, topical use of imiquimod	The number of and the size of tumors decreased
Donati, M. et al. (6)	F/62	Kidney transplantation for end-stage renal disease	Cyclosporine, Azathioprine, Cortisone/Tacrolimus, Azathioprine, Cortisone		Multiple popular keratotic lesions on face and arm and one infiltrative nodular lesion on posterior part of right thigh	IHC:MLH-1(+), MSH2(+); Germ-line mutation analysis: MSH6-eson 1 (c116G>A)	Sebaceous carcinoma, keratoacanthoma	Change form tacrolimus to everolimus	The original tumor disappeared without new one
Shaw, K.C. et al. (7)	M/65	Heart transplantation	Tacrolimus, Mycophenolate mofetil	Mother and mother’s aunt had colon adenocarcinoma	Erythematous nodular papule on right middle back	IHC: MSH-6(−); Germ-line mutation analysis: MSH6-exon2 (c.432delC)	Sebaceous carcinoma		Later developed sebaceous adenoma, squamous cell carcinoma with keratoacanthoma features, squamous cell carcinoma with pseudoadenoid differentiation, non-small cell lung adenocarcinoma and multiple myeloma
Ponti, G. et al. (8)	M/49	Kidney transplantation, colon adenoma	Tacrolimus, prednisone	One sister has colon cancer, while another sister and one niece have endometrial cancer	Lesions on the face and lumbar spine		Sebaceous adenoma, basal cell carcinoma, keratoacanthoma		

M, male; F, female; IHC, immunohistochemical; MTS, Muir–Torre syndrome.

The key to diagnosing MTS is to have single or multiple cutaneous sebaceous skin tumors along with at least one visceral malignant tumor (1). Sebaceous adenomas are the sebaceous neoplasms most closely associated with MTS, while sebaceous carcinomas are more rare, and, unlike sporadic sebaceous carcinomas, are less aggressive, have a slower course, and mostly arise on the trunk (14). Colorectal cancer is the most common visceral neoplasm associated with MTS, followed by endometrial cancer, urinary tract neoplasms, upper gastrointestinal cancer, and pancreatic cancer (15).

There are no clear guidelines for the clinical management of MTS, so deciding whether a patient warrants MTS screening remains challenging. Most experts recommend IHC and MSI analysis for MMR defects in all sebaceous gland neoplasms, even without MTS-related personal or family history (16). On the basis of the Mayo MTS risk score algorithm, we concluded that patients with a score ≥2 or a personal and/or family history should directly undergo germline mutation analysis, while all others should undergo IHC and MSI testing.

As the routine test for MTS, individual IHC analysis provides a low level positive predictive value of MMR (MSH2: 55%, MLH1: 88%, MSH6: 67%), while combined loss of certain gene products provides a positive predictive value of 100%, such as MLH1/MSH6 and MLH1/MSH2/MSH6 (17). Furthermore, studies have found that the sensitivity and specificity of IHC analysis for MTS in sebaceous gland neoplasms were lower than in colon or endometrial tumors (18). So, it is recommended that patients complicated with colon or endometrial tumors be evaluated by these tumor types. Furthermore, if MSI testing is positive and/or IHC analysis shows loss of MMR protein(s), germline mutation analysis should be performed, which is an important way to confirm clinical presentations, helping differentiate MTS from Gardner Syndrome, Peutz–Jeghers’s syndrome, Cowden syndrome, Brooke–Spiegler syndrome, basal cell nevus syndrome, Ferguson–Smith syndrome, and tuberous sclerosis (14). However, given the possibility of unrecognized MTS II patients, patients with a high suspicion of MTS should undergo both germline mutation analysis and tumor surveillance, even in the absence of MSI. It should be recognized that detecting a deleterious germline mutation in a particular MMR gene in a patient with a history of a skin tumor can be used to diagnose MTS, even in the absence of visceral organ malignancy, as the occurrence of sebaceous gland neoplasms can present before (22%), simultaneously (6%), or after (56%) (19) visceral malignancies have developed. Also, the patient and their relatives should be followed up closely to discover new-onset cancers early, especially colorectal cancer (20–23). Interestingly, the presence of any DNA damage response alteration was associated with a higher response rate to PD-1 treatment and longer progression-free and overall survival (24), indicating the potentially important role that alterations in DNA damage response and repair genes play in antitumor treatments.

In conclusion, we report the case of a kidney transplant recipient with sebaceous carcinoma concurrent with sigmoid adenocarcinoma, consistent with a clinical diagnosis of MTS. The findings presented herein add to our understanding of the screening process for MTS and indicate a possible relationship between kidney transplantation and MTS progression, suggesting the importance of further preventing the development of latent visceral tumors in kidney transplant recipients.

## Data Availability Statement

The original contributions presented in the study are included in the article/supplementary material. Further inquiries can be directed to the corresponding author.

## Ethics Statement

Written informed consent was obtained from the individual(s) for the publication of any potentially identifiable images or data included in this article.

## Author Contributions

JRB and JQF put forward the content of the paper. YFF wrote the manuscript. YFF, JQF, and JRB reviewed literature and clinical data. All authors contributed to the article and approved the submitted version.

## Conflict of Interest

The authors declare that the research was conducted in the absence of any commercial or financial relationships that could be construed as a potential conflict of interest.
